# Trends and Risk Factors of Pediatric Venous Thromboembolism in Spain: A Nationwide Study from 2016 to 2023

**DOI:** 10.3390/jcm14113950

**Published:** 2025-06-03

**Authors:** José Antonio Rueda-Camino, Gema Sabrido-Bermúdez, Raquel Barba-Martín

**Affiliations:** 1 Venous Thromboembolic Disease Unit, Internal Medicine, Hospital Universitario Rey Juan Carlos, C/Gladiolo s/n, 28933 Móstoles, Spain; raquel.barba@hospitalreyjuancarlos.es; 2 Health Research Institute Fundación Jiménez Díaz, 28040 Madrid, Spain; 3 Pediatric Hematology, Hospital Universitario Rey Juan Carlos, C/Gladiolo s/n, 28933 Móstoles, Spain

**Keywords:** pediatrics, venous thromboembolism, epidemiology

## Abstract

**Background:** The incidence of pediatric venous thromboembolism (VTE) in Spain has not been well studied. **Methods**: Using an administrative database comprising nationwide data on hospital discharges, we estimated the annual crude, age-specific, and age-standardized incidence of pediatric VTE in Spain from 2016 to 2023. Time trends were analyzed using joinpoint regression. Risk factors, complications, and in-hospital mortality were also assessed. **Results**: A total of 6510 cases were identified, and 45.1% were women; the median age was 3 years (p25–p75: 0–13). The prevalence of cancer, intravascular device use, and chronic complex conditions decreased over the study period, while liver disease and surgery rates increased. COVID-19 emerged as a risk factor in the last four years. The presence of chronic conditions, congenital heart disease, and intravascular devices was significantly higher in neonates. Contraceptive use was observed only in adolescents. Overall incidence of VTE decreased from 2016 to 2018 (annual percent change, APC −10.1%, *p* = 0.234), but significantly increased from 2018 to 2023 (APC 7.9%, *p* = 0.018). The occurrence of hemorrhage significantly increased: 3.9% in 2016 vs. 6.5% in 2023 (*p* = 0.014). Bleeding risk increased with age (2.3% in <1 year vs. 7.4% in 15–18 years, *p* < 0.001). In-hospital mortality remained stable (2.41% in 2016 vs. 2.25% in 2023, *p* = 0.493). Mortality was higher in neonates (3.95%) and adolescents aged 15–18 years (3.05%) compared to other age groups (*p* < 0.001). **Conclusions**: The incidence of pediatric VTE in Spain has increased in recent years, while in-hospital mortality has remained stable.

## 1. Introduction

The overall incidence of venous thromboembolism (VTE) in the pediatric population is extremely low, ranging between 0.7 and 4.9 cases per 100,000 children [1,2,3,4,5,6]. However, this risk rises dramatically in hospitalized children, increasing by 100- to 1000-fold to exceed 58 cases per 10,000 hospital admissions [7]. The reported incidence varies depending on the type of study and the types of thrombosis included, as some studies also consider cerebral venous sinus thrombosis, superficial thrombosis, or arterial thrombosis, while others do not [8]. The most frequently affected age groups for VTE are neonates and adolescents, reflecting the typical patterns of underlying conditions and medical interventions.

The presence of a central venous access device (CVAD) [7,9] is a common precipitating factor for venous thromboembolism (VTE) in both neonates and older children. Inherited thrombophilia contributes significantly to the risk of both provoked and unprovoked VTE. The most frequent hereditary risk factors include deficiencies in protein C, protein S, and antithrombin, as well as the factor V Leiden and prothrombin G20210A mutations [10]. Acquired risk factors include malignancies, infections, obesity, congenital heart disease, nephrotic syndrome, and chronic inflammatory disorders [11]. Pediatric oncology patients—especially those with acute lymphoblastic leukemia—are particularly vulnerable due to disease- and treatment-related prothrombotic mechanisms [5,12]. As the prevalence of chronic conditions and obesity increases among children, so does the incidence of VTE, underscoring the need for greater awareness and targeted preventive measures.

Differences in thrombosis patterns between children and adults can be attributed to developmental variations in the hemostatic system [11]. In neonates and infants, a lower capacity for thrombin generation, the enhanced inhibitory activity of alpha-2-macroglobulin, and the antithrombotic properties of the vessel wall offer natural protection against clot formation. Furthermore, children are less frequently exposed to endothelial damage from conditions common in adults, such as hypertension, diabetes, hypercholesterolemia, or acquired prothrombotic factors like smoking and antiphospholipid antibodies [13]. Nonetheless, chronic pediatric conditions such as obesity and diabetes can lead to early endothelial dysfunction and elevate the risk of cardiovascular complications from an early age [7,14]. Furthermore, it seems that there are some differences among children depending on their age as VTE peaks in neonates (<1 year) and adolescents (15–18 years) [1,3]. Regarding sex, it is not clear whether it is a risk factor or not, as previous reports showed conflicting results [2,6].

Although older reports indicated a stable incidence of pediatric VTE, more recent studies showed an important increase. In Canada [4], pulmonary embolism incidence in pediatric population rose from 2.1 cases per million children in 2020 to 2.9 cases per million children in 2022. Similar results were observed in the United States of America, where incidence of VTE in hospitalized children increased from 34 to 58 cases per 10,000 admissions in the 2001–2007 period [7]. Data for trends in pediatric VTE incidence in the European region in recent years are lacking.

Venous thromboembolism in children can lead to serious complications, including pulmonary embolism [15], stroke due to paradoxical embolism, organ dysfunction, infections, post-thrombotic syndrome (PTS), pain, and even loss of venous access. It is also associated with increased hospital stays and higher healthcare costs [16]. Although the estimated mortality rate [2] in pediatric VTE is around 2.2%, this figure likely underrepresents the true burden of the disease.

We find it relevant to examine the diagnosed cases of VTE requiring hospitalization in children in Spain over recent years to determine whether there has been an increase in incidence, as reported in other countries. Additionally, we aim to identify the associated risk factors, mortality, and hemorrhagic complications.

## 2. Materials and Methods

### 2.1. Data Source and Patient Selection

We utilized the hospital discharge database of the National Health System (NHS) in our country (*Registro de Atención Especializada–Conjunto Mínimo Básico de Datos, CMBD*) to conduct a retrospective study on pediatric VTE incidence time trends in Spain from 2016 to 2023. The RAE-CMBD is a pseudonymized administrative database encompassing all hospital discharges from public (NHS) and private healthcare facilities across the country. Data in the database are coded according to the International Classification of Diseases (ICD-10-ES) and include patient-level information such as demographics and hospitalization characteristics, including primary and secondary diagnoses, procedures performed during the stay, reasons for admission, length of stay, discharge outcomes, and hospital costs. As reporting their data to the RAE-CMBD is a legal requirement for all Spanish hospitals, irrespective of their administration modality, it contains information on the totality of hospital admissions.

The analysis included all patients discharged in the country who were admitted either as emergency or elective cases, were under 18 years old, and presented with a diagnosis of venous thromboembolic disease at admission. Patients were included if the diagnosis was listed as either the primary or a secondary diagnosis. The presence of comorbidities was assessed as previously described [17].

Diagnoses of thromboembolic disease were identified by the presence of relevant ICD-10-ES indicating VTE, including the following: deep vein thrombosis **(DVT) of the lower extremities**: I80.1x, I80.2x, I80.3x, I80.8x, I80.9x, I82.4x, **DVT of the upper extremities**: I82.6x, I82.A1x, I82.B1x, I82.C1x, **pulmonary embolism** (PE): I26.0, I26.02, I26.09, I26.92, I26.93, I26.94, I26.99, **superficial thrombophlebitis**: I80.0x, **portal vein thrombosis** (PVT) **and hepatic vein thrombosis**: I81, K75.1, **renal vein thrombosis** I82.3, **obstetric-associated thromboembolism**: O22.2x, O22.3x, O22.9x, O87.0, O87.1, O88.21x, O88.22, O88.23, O88.81x, O88.82, O88.83, **abortion-related thrombosis**: O03.2, O03.35, O03.7, O03.85, O04.7, O04.85, O07.2, O08.2, O08.7, or **other venous thromboses** (e.g., unspecified or multiple sites): I82.2x, I82.210, I82.220, I82.290, I82.81x, I82.1, I82.890, I82.90, I82.60x, I82.62. This approach has been validated previously [17,18,19]. To identify whether hospitalized pediatric patients had at least one coexisting complex chronic condition (CCC) [20], we applied a classification system based on ICD-10-ES codes. This previously validated approach categorizes chronic conditions into ten mutually exclusive groups: neuromuscular, cardiovascular, respiratory, renal, gastrointestinal, hematologic or immunodeficiency-related, metabolic, other congenital or genetic disorders, and malignancy. We also identified patients with any hemorrhage by selecting the patients having any of the following codes in any position: D62, D68.3, D69.8, D69.9, H11.3, H21.0, H31.2, H31.3, H35.6, H43.1, H92.2, I23.0, I60.x, I61.3, I61.4, I61.5, I61.6, I61.7, I61.8, I61.9, I85.0, J94.2, K22.6, K25.0, K25.2, K25.4, K25.6, K26.0, K26.2, K26.4, K26.6, K27.0, K27.2, K27.4, K27.6, K28.0, K28.2, K28.4, K28.6, K29.0, K62.5, K66.1, K76.2, K92.0, K92.1, K92.2, M25.0, N02.x, N42.1, N92.x, N93.0, N93.8, N93.9, N95.0, R04.x, R31.x, R58, S06.4, S06.5, S06.6, S26, S27.1, or T79.2.

Population data were obtained from the National Statistics Institute [21]. 

### 2.2. Statistical Analysis

Patients were divided into the following age groups: neonates (<1 year), children (1–4 and 5–9 years), and adolescents (10–14 and 15–18 years). This division was made to ensure comparability with previous studies.

For each year during the study period, the proportion of medical conditions was presented with absolute and relative frequencies. Age and length of stay were expressed as medians and interquartile ranges. We tested for a monotonic trend in risk factor prevalence over time using the Cochran–Armitage test for categorical variables and the Kruskal–Wallis test for age and length of stay. Additionally, the differences in risk factor prevalence between age groups were described with counts and percentages and tested with the chi-squared test.

The annual crude incidence rate of VTE was calculated by dividing the number of pediatric patients with VTE each year by the resident population under 18 years old for the same year; 95% confidence intervals were calculated using the exact method. To compare incidence rates across different years while accounting for variations in age group distributions, an annual standardized incidence rate was estimated using the direct method, with the age distribution of the entire period serving as the reference population. Time trends were evaluated with joinpoint regression version 5.0.2 (Surveillance Research Program, National Cancer Institute, USA). The analyses were also conducted separately for each sex.

In-hospital mortality was estimated by dividing the number of cases discharged as deceased by the total number of VTE discharges.

Data analysis was conducted using Stata/BE 17.0 (StataCorp LLC, College Station, TX, USA). The data were obtained following the operational procedures of the Spanish Ministry of Health. In accordance with current Spanish legislation and due to the use of pseudonymized data, informed consent was not required. The manuscript was written following the STROBE statement recommendations.

## 3. Results

**Figure 1:**
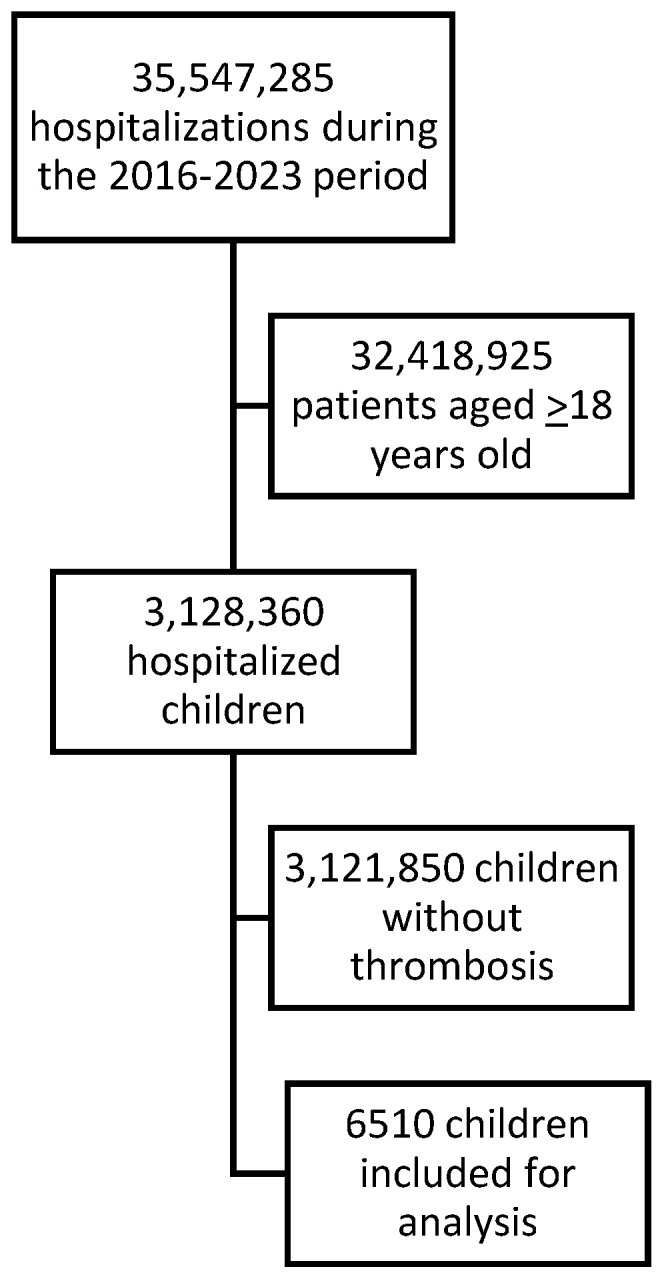
Flow chart of patients.

During the 2016–2023 period, 6510 pediatric patients were discharged from Spanish hospitals following a VTE episode, including those who developed thrombosis during hospitalization. Of these, 2935 (45.1%) were women. The median age was 3 years. Table 1 presents the main characteristics of the study population and Figure 1 presents the flow chart of patients. The more frequent form of VTE at presentation was lower limb DVT.

The prevalence of cancer, the use of intravascular devices, and the presence of chronic complex conditions showed a decreasing trend during the study period, whereas liver disease and surgery increased. In the last four years, COVID-19 emerged as a risk factor.

As shown in Table 2, boys constituted the majority of population except for older adolescents where girls are the majority of the sample. The presence of CCC, CHD, and intravascular devices was significantly higher in neonates, in whom liver disease, CKD, and cancer was significantly less frequent. The use of contraceptives was, as expected, only observed in adolescents. Also, 15 cases of pregnancy-related VTE were observed (9 patients aged 18, 4 aged 17, and 2 aged 16 years old).

The number of cases of each type of thrombosis is shown in Table 3.

**Figure 2:**
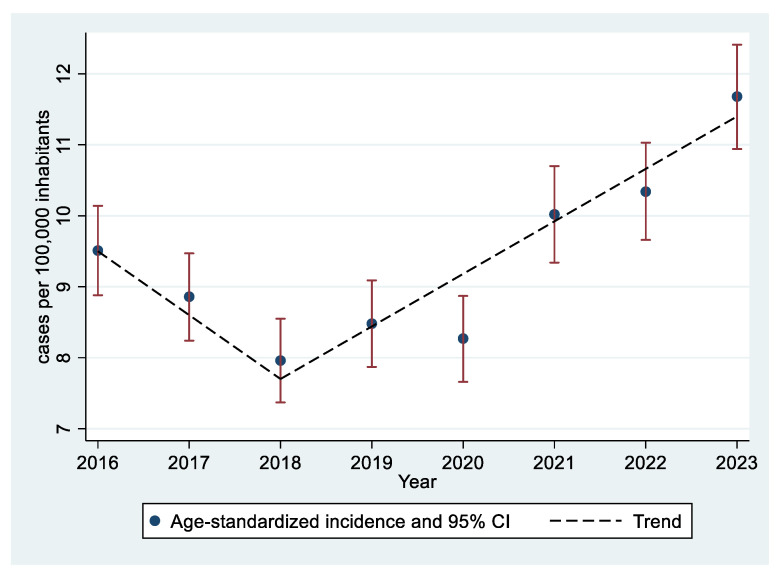
Age-standardized VTE incidence.

The overall incidence of VTE exhibited a biphasic pattern: from 2016 to 2018, it showed a non-significant decreasing trend with an annual percent change (APC) of −10.1% (*p* = 0.234); from 2018 to 2023, it significantly increased (APC 7.9%, *p* = 0.018). Annual crude, age-specific, and standardized rates are shown in Table 4 and Figure 2.

A post hoc analysis excluding patients diagnosed with COVID-19 showed similar results. A biphasic pattern was observed: the age-standardized incidence fell from 9.51 in 2016 to 7.96 cases per 100,000 children in 2018 (APC −10.0%, *p* = 0.263) and increased from 2018 onwards to 11.43 cases per 100,000 children in 2023 (APC 7.1%, *p* = 0.029).

**Figure 3:**
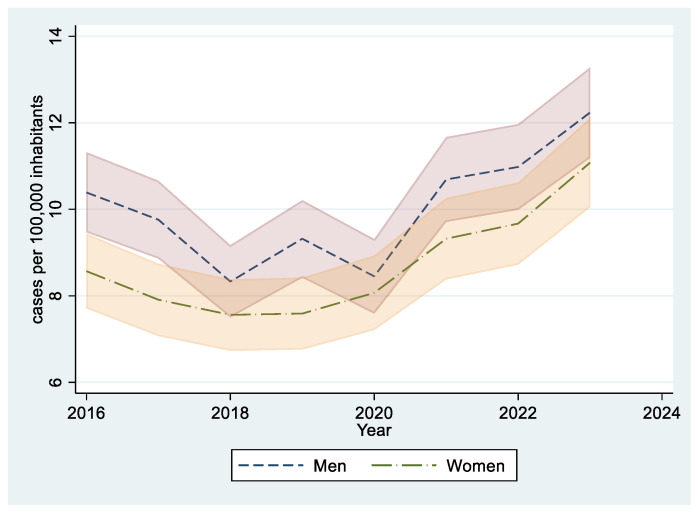
Incidence of pediatric VTE in men and women. Sex-specific incidences are shown alongside their 95% confidence interval.

Incidence was higher among men, as depicted in Figure 3. Detailed analysis of incidence by sex is shown in Table 5 and Table 6. In men, age-standardized incidence followed a biphasic pattern, replicating the results of the whole population: from 2016 to 2018, it showed a non-significant decreasing trend (APC −11.4, *p* = 0.326). From 2018 to 2023, it significantly increased, with an APC of 7.6% (*p* = 0.050).

During the study period, 336 (5.2%) bleedings were registered. The proportion of patients suffering a hemorrhage significantly increased from 3.9% in 2016 to 6.5% in 2023 (*p* = 0.014). The bleeding risk increased with age: 2.3% (<1 year), 6.0% (1–4 years), 6.9% (5–9 years), 7.6% (10–14 years), and 7.4% (15–18 years). These differences were statistically significant (*p* < 0.001). According to thrombosis site, bleedings occurred as follows: 120 (35.7%) in patients with lower limb DVT, 99 (29.5%) in patients with portal vein thrombosis, 63 (18.8%) in patients with pulmonary embolism, 27 (8%) in patients with renal vein thrombosis, 9 (2.7%) in patients with upper limb DVT, 6 (1.8%) in patients with superficial vein thrombosis, and 12 (3.6%) in patients with other sites thrombosis.

Regarding mortality, 188 patients died, resulting in an overall in-hospital mortality rate of 2.89%. In-hospital mortality remained stable throughout the study period (2.41% in 2016 vs. 2.25% in 2023, *p* = 0.493). By age group, mortality was significantly higher in children under one year (3.95%) and adolescents aged 15 to 18 years (3.05%) compared to other age groups: 2.18% in children aged 1–4 years, 2.12% in children aged 5–9 years, and 1.22% in adolescents aged 10–14 years (*p* < 0.001). Due to the nature and structure of data in the registry, the cause of death could not be affirmed.

## 4. Discussion

This study established the incidence of pediatric venous thromboembolism (VTE) in Spain using a nationwide administrative database that includes all hospitalized patients over a five-year period. Given the severity of this condition, it is reasonable to assume that most cases—particularly those involving pulmonary embolism—are captured in this dataset. Based on these data, the estimated incidence of pediatric VTE in Spain is approximately 10 cases per 100,000 children per year, with an upward trend observed in recent years. This study shows the most up-to-date and comprehensive estimates for Spain, and the European region by extension.

In our series, there was an overall male predominance, except in the adolescent group aged 15–18 years, where females were more prevalent. Other studies have shown mixed findings, with some reporting a balanced sex distribution [1,3,6] and others a female predominance [1,2,4,5,8]. In general, studies that exclude neonates or involve populations with a higher mean age tend to show a greater proportion of female patients. This is likely explained by the proportion of female patients who develop thrombosis related to hormonal factors [1,4], such as the use of oral contraceptives, pregnancy, or pregnancy loss.

A high percentage of our patients had at least one risk factor that may help explain the presence of thromboembolic disease, with variations observed across different age groups. Approximately 15% had congenital heart disease, around 10% had an underlying malignancy, 3% had thrombophilia, 5% sepsis, and more than 70% had one or more coexisting complex chronic conditions. In addition, the presence of a vascular device is reported in one third of the patients, although in those under one year of age, the proportion rises to two-thirds, as reported in other studies [3,6]. This is consistent with previous studies [3,4,7], which describe that VTE in pediatric patients is rarely idiopathic. Another comorbidity whose incidence has increased during the study period is liver disease, a fact that could be explained by the increasing incidence over the past several years of non-alcoholic steatohepatitis [22].

The incidence observed in our study is slightly higher than that reported in previous publications [1,2,3,4,5,8], which may be explained by several factors. First, our analysis includes superficial and atypical site thromboses conditions that have not always been included in other studies, although cases of cerebral venous sinus thrombosis were excluded. Second, as our data source comprises a nationwide registry that captures all hospital admissions, it is likely that most cases of venous thromboembolism were identified, minimizing the risk of underreporting. This comprehensive coverage may account for the higher incidence rates observed compared to other registries [2,8] that may miss cases due to more limited data collection.

In line with the studies of Krmpotic [4] and Raffini [7], we observed a significant increase in the incidence of pediatric VTE during the last five years. In our case, it may be partially explained by the COVID-19 pandemic, as the disease is a well-known risk factor for thrombosis [23]. Our data support this notion as the APC for the 2018–2023 period is slightly lower after eliminating the cases diagnosed with COVID-19. The increase in surgical procedures and prevalence of liver disease may have counterbalanced the decrease in use of intravascular devices and cancer frequency.

As observed in previous reports [1,2,8], a bimodal distribution in the incidence of thrombosis is evident, with a prominent peak during the first year of life—primarily associated with the use of central venous catheters and congenital heart disease—and a less pronounced increase during adolescence. In this latter age group, risk factors such as the use of oral contraceptives or pregnancy may contribute to this; however, in our series, very few cases could be directly linked to these circumstances.

Bleeding is a complication recorded in 5% of patients, with an upward trend observed in recent years. The frequency is similar to that reported in other studies [3,14]. However, due to the different methodologies used to capture this complication, making direct comparisons across studies is challenging.

During the study period, 188 patients died, resulting in an overall in-hospital mortality rate of 2.89%. In-hospital mortality was significantly higher among infants under 1 year of age (3.95%) and adolescents aged 15–18 years (3.05%) compared to other age groups. These findings are consistent with previous reports that have documented a wide range of mortality rates in pediatric VTE, from 0.5% to 21% [2,5,24,25]. Our mortality rate falls within the lower end of this spectrum, possibly reflecting improvements in the management of underlying comorbidities frequently associated with VTE. As reported by other authors, mortality is often higher in the youngest patients, likely due to the frequent coexistence of critical illness at the time of VTE diagnosis [2,3]. While our dataset does not allow us to determine whether VTE was the direct cause of death, these findings reinforce that pediatric patients with VTE often represent a clinically vulnerable population.

In light of our results, various prevention strategies could be considered. Firstly, given the increasing trend in incidence, programs should be implemented to raise the level of awareness about this health issue among professionals who care for children. Secondly, risk identification systems integrated into the electronic health record (most likely with the support of AI tools) should be implemented to help detect which patients are at the highest risk, thereby allowing greater efforts to be directed towards them. Lastly, given that the use of intravascular devices remains one of the primary risk factors, adopting strategies to reduce the associated risk seems reasonable. This includes limiting their use to cases where other drug administration routes are not possible, choosing the type of catheter and insertion site with the lowest risk, and reviewing daily during hospitalization whether the indication for maintaining the device is still valid, among other measures.

This study has several limitations, many of which are inherent to research based on administrative datasets [26]. The findings are subject to the accuracy and completeness of data entry, as well as potential confounding factors. Cases of VTE in patients who were not hospitalized or who died from pulmonary embolism before admission may not have been captured, as outpatient data were not available. Nevertheless, we are confident that all cases recorded within the hospital setting have been comprehensively included using nationwide, population-based data. Although the possibility of miscoding cannot be ruled out, the validity of VTE diagnoses in this dataset has been previously assessed against medical records, demonstrating a positive predictive value exceeding 90% [17]. Another limitation is the lack of granularity in the dataset regarding symptoms, diagnostic tests, or treatment. Also, there are no data regarding severity, as the severity indexes provided by the RAE-CMBD refer to resource consumption and not the clinical tableau. Nevertheless, due to the fact that the database covers all admissions, it is very representative of the pediatric VTE Spanish population.

## 5. Conclusions

In conclusion, this is the first study to provide nationwide estimates of pediatric VTE incidence and in-hospital mortality in a European country in recent years. Although overall incidence was relatively low, we observed a decline in the number of cases during the initial two years of the study, followed by a progressive increase, which may be partly influenced by the COVID-19 pandemic or other evolving clinical and diagnostic factors. While the overall risk of death was lower than in some international reports, it remains notably higher than the background mortality in the general pediatric population. These findings underscore the need for continued surveillance of VTE trends in children and for future studies to refine prevention and management strategies, particularly for high-risk age groups.

## Figures and Tables

**Table 1:** Main characteristics of the population.

	Overall	2016	2017	2018	2019	2020	2021	2022	2023	*p* ^(*)^
**Women**	2935 (45.1)	381 (43.6)	346 (43.3)	323 (45.9)	321 (43.4)	338 (47.5)	380 (45.5)	398 (45.6)	448 (45.9)	0.174
**Age (years)**	3 (0–13)	1 (0–11)	3 (0–12)	3 (0–13)	2 (0–12)	3 (0–14)	4 (0–14)	4 (0–14)	7 (0–13)	0.165
**Previous VTE**	65 (1.0)	8 (0.9)	3 (0.4)	6 (0.9)	11 (1.5)	7 (1)	6 (0.7)	9 (1)	15 (1.5)	0.112
**Cancer**	674 (10.4)	129 (14.8)	102 (12.8)	59 (8.4)	49 (6.6)	75 (10.5)	85 (10.2)	66 (7.6)	109 (11.2)	0.002
**COVID-19**	95 (1.5)	0 (0)	0 (0)	0 (0)	0 (0)	2 (0.3)	26 (3.1)	47 (5.4)	20 (2)	<0.001
**Liver disease**	567 (8.7)	44 (5)	32 (4)	42 (6)	75 (10.1)	66 (9.3)	69 (8.3)	91 (10.4)	148 (15.2)	<0.001
**CKD**	155 (2.4)	17 (1.9)	31 (3.9)	16 (2.3)	14 (1.9)	15 (2.1)	16 (1.9)	25 (2.9)	21 (2.2)	0.542
**Connective tissue disease**	50 (0.8)	6 (0.7)	7 (0.9)	9 (1.3)	5 (0.7)	4 (0.6)	5 (0.6)	3 (0.3)	11 (1.1)	0.784
**Sepsis**	344 (5.3)	41 (4.7)	54 (6.8)	37 (5.3)	40 (5.4)	40 (5.6)	28 (3.3)	51 (5.8)	53 (5.4)	0.683
**CHD**	978 (15.0)	117 (13.4)	136 (17)	126 (17.9)	111 (15)	116 (16.3)	126 (15.1)	122 (14)	124 (12.7)	0.087
**Sickle cell disease**	41 (0.6)	6 (0.7)	2 (0.3)	5 (0.7)	4 (0.5)	5 (0.7)	5 (0.6)	6 (0.7)	8 (0.8)	0.394
**Nephrotic syndrome**	44 (0.7)	5 (0.6)	10 (1.3)	5 (0.7)	5 (0.7)	8 (1.1)	1 (0.1)	4 (0.5)	6 (0.6)	0.183
**Transplantation**	130 (2.0)	22 (2.5)	16 (2)	9 (1.3)	14 (1.9)	18 (2.5)	15 (1.8)	14 (1.6)	22 (2.3)	0.750
**Thrombophilia**	154 (2.4)	23 (2.6)	22 (2.8)	16 (2.3)	13 (1.8)	23 (3.2)	15 (1.8)	16 (1.8)	26 (2.7)	0.532
**Overweight or obesity**	112 (1.7)	12 (1.4)	12 (1.5)	9 (1.3)	10 (1.4)	21 (2.9)	12 (1.4)	13 (1.5)	23 (2.4)	0.122
**Intravascular device**	2083 (32.0)	362 (41.5)	300 (37.5)	230 (32.7)	222 (30)	227 (31.9)	279 (33.4)	239 (27.4)	224 (23)	<0.001
**Contraceptive use**	34 (0.5)	5 (0.6)	5 (0.6)	7 (1)	2 (0.3)	4 (0.6)	1 (0.1)	4 (0.5)	6 (0.6)	0.435
**CCC**	4625 (71.0)	662 (75.8)	597 (74.7)	497 (70.7)	537 (72.7)	512 (71.9)	579 (69.3)	574 (65.8)	667 (68.3)	<0.001
**Surgery**	71 (1.1)	2 (0.2)	4 (0.5)	3 (0.4)	8 (1.1)	13 (1.8)	9 (1.1)	19 (2.2)	13 (1.3)	<0.001
**Length of stay (days)**	10 (5–23)	8 (4–22)	10 (5–24)	10 (5–22)	11 (5–23)	10 (5–26)	9 (5–22)	10 (5–26)	9 (4–21)	0.909

Categorical variables are shown as [*n*, (%)] and continuous variables as median (p25-p75). CCC: chronic complex condition; CHD: congenital heart disease; CKD: chronic kidney disease; VTE: venous thromboembolism. (*) Cochran–Armitage or Kruskal–Wallis test, as appropriate.

**Table 2:** Distribution of risk factors across age groups.

	<1 Year	1–4 Years	5–9 Years	10–14 Years	15–18 Years	*p* ^(*)^
**Women**	1105 (43.6)	411 (42.6)	343 (45.6)	420 (42.8)	656 (51.3)	<0.001
**Previous VTE**	7 (0.3)	21 (2.2)	6 (0.8)	7 (0.7)	24 (1.9)	<0.001
**Cancer**	28 (1.1)	187 (19.4)	122 (16.2)	189 (19.2)	148 (11.6)	<0.001
**COVID-19**	21 (0.8)	15 (1.6)	15 (2)	9 (0.9)	35 (2.7)	<0.001
**Liver disease**	37 (1.5)	144 (14.9)	114 (15.1)	166 (16.9)	106 (8.3)	<0.001
**CKD**	14 (0.6)	42 (4.4)	33 (4.4)	25 (2.5)	41 (3.2)	<0.001
**Connective tissue disease**	5 (0.2)	6 (0.6)	8 (1.1)	13 (1.3)	18 (1.4)	<0.001
**Sepsis**	144 (5.7)	50 (5.2)	30 (4)	51 (5.2)	69 (5.4)	0.538
**CHD**	744 (29.4)	85 (8.8)	67 (8.9)	41 (4.2)	41 (3.2)	<0.001
**Sickle cell disease**	0 (0)	4 (0.4)	14 (1.9)	8 (0.8)	15 (1.2)	<0.001
**Thrombophilia**	20 (0.8)	19 (2)	23 (3.1)	34 (3.5)	58 (4.5)	<0.001
**Nephrotic syndrome**	6 (0.2)	21 (2.2)	6 (0.8)	3 (0.3)	8 (0.6)	0.788
**Transplantation**	1 (0)	34 (3.5)	24 (3.2)	26 (2.6)	45 (3.5)	<0.001
**Surgery**	43 (1.7)	4 (0.4)	5 (0.7)	10 (1)	9 (0.7)	0.007
**Overweight or obesity**	1 (0)	1 (0.1)	5 (0.7)	36 (3.7)	69 (5.4)	<0.001
**Intravascular device**	1431 (56.5)	194 (20.1)	121 (16.1)	145 (14.8)	192 (15)	<0.001
**Contraceptive use**	0 (0)	0 (0)	0 (0)	2 (0.2)	32 (2.5)	<0.001
**CCC**	2089 (82.5)	673 (69.8)	496 (65.9)	650 (66.2)	717 (56.1)	<0.001

Variables are shown as [n, (%)]. CCC: chronic complex condition [20]; CHD: congenital heart disease; CKD: chronic kidney disease; VTE: venous thromboembolism. (*) Chi-squared test.

**Table 3:** Yearly distribution of any type of venous thromboembolism.

	Overall	2016	2017	2018	2019	2020	2021	2022	2023
**Lower limb DVT**	3460	501	421	405	444	390	433	423	443
**PE**	784	62	81	75	89	99	112	136	130
**Upper limb DVT**	189	45	36	10	8	14	25	24	27
**Superficial**	104	14	20	6	5	14	11	16	18
**Renal**	187	34	35	29	14	12	20	17	26
**Portal**	1014	107	97	86	104	113	129	169	209
**Other**	772	110	109	92	75	70	106	87	123

Absolute counts.

**Table 4:** Annual crude, age-specific, and age-standardized incidence rates of pediatric VTE.

Year	Population	Cases	Crude Rate (CI 95%)	<1 Year	1–4 Years	5–9 Years	10–14 Years	15–18 Years	Age-Standardized Rate (CI 95%)
**2016**	8,736,116	873	9.99 (9.34 a 10.68)	102.46	7.26	3.38	4.37	8	9.51 (8.88–10.14)
**2017**	8,736,111	799	9.15 (8.52 a 9.8)	84.3	7.06	4.86	3.62	7.81	8.86 (8.24–9.47)
**2018**	8,736,859	703	8.05 (7.46 a 8.66)	78.13	5.56	3.62	3.3	8.06	7.96 (7.37–8.55)
**2019**	8,733,132	739	8.46 (7.86 a 9.09)	90.58	6.27	2.53	4.32	7.49	8.48 (7.87–9.09)
**2020**	8,699,072	712	8.18 (7.59 a 8.81)	80.34	6.05	3.09	4.2	8.1	8.27 (7.66–8.87)
**2021**	8,593,001	836	9.73 (9.08 a 10.41)	100.47	7.89	3.11	5.13	9.53	10.02 (9.34–10.7)
**2022**	8,584,406	872	10.16 (9.49 a 10.86)	88.23	9.58	4.84	5.5	9.27	10.34 (9.66–11.03)
**2023**	8,562,979	976	11.4 (10.69 a 12.14)	77.48	11.04	7.15	9.14	8.59	11.68 (10.94–12.41)

Results showed as cases per 100,000 children. CI: confidence interval.

**Table 5:** Annual crude, age-specific, and age-standardized incidence rates of pediatric VTE (boys).

Year	Population	Cases	Crude Rate (CI 95%)	<1 Year	1–4 Years	5–9 Years	10–14 Years	15–18 Years	Age-Standardized Rate (CI 95%)
**2016**	4,498,422	492	10.94 (9.99 a 11.95)	113.07	8.09	3.79	4.62	8.48	10.39 (9.47–11.32)
**2017**	4,497,516	453	10.07 (9.17 a 11.04)	87.15	9.18	5.59	4.21	7.91	9.76 (8.86–10.66)
**2018**	4,497,683	380	8.45 (7.62 a 9.34)	84.18	6.78	3.75	3.4	7.31	8.33 (7.5–9.17)
**2019**	4,496,846	418	9.3 (8.43 a 10.23)	97.15	8.32	3	4.66	7.32	9.32 (8.42–10.21)
**2020**	4,478,756	374	8.35 (7.53 a 9.24)	87.68	5.82	3.04	4	8.07	8.45 (7.59–9.31)
**2021**	4,424,081	456	10.31 (9.38 a 11.3)	121.2	8.25	2.67	5.71	8.11	10.69 (9.71–11.67)
**2022**	4,419,345	474	10.73 (9.78 a 11.74)	101.96	10.44	5.75	5.37	7.89	10.98 (9.99–11.97)
**2023**	4,409,571	528	11.97 (10.97 a 13.04)	78.22	10.41	6.77	12.01	8.28	12.24 (11.19–13.28)

Results showed as cases per 100,000 inhabitants. CI: confidence interval.

**Table 6:** Annual crude, age-specific, and age-standardized incidence rates of pediatric VTE (girls).

Year	Population	Cases	Crude Rate (CI 95%)	<1 Year	1–4 Years	5–9 Years	10–14 Years	15–18 Years	Age-Standardized Rate (CI 95%)
**2016**	4,237,694	381	8.99 (8.11 a 9.94)	91.21	6.36	2.94	4.1	7.49	8.57 (7.71–9.44)
**2017**	4,238,595	346	8.16 (7.33 a 9.07)	81.28	4.81	4.08	2.99	7.7	7.91 (7.07–8.74)
**2018**	4,239,176	323	7.62 (6.81 a 8.5)	71.72	4.27	3.47	3.18	8.87	7.56 (6.73–8.38)
**2019**	4,236,286	321	7.58 (6.77 a 8.45)	83.64	4.1	2.04	3.95	7.67	7.59 (6.76–8.42)
**2020**	4,220,316	338	8.01 (7.18 a 8.91)	72.59	6.29	3.14	4.41	8.14	8.07 (7.21–8.93)
**2021**	4,168,920	380	9.12 (8.22 a 10.08)	78.55	7.51	3.57	4.51	11.03	9.32 (8.38–10.26)
**2022**	4,165,061	398	9.56 (8.64 a 10.54)	73.61	8.66	3.88	5.63	10.74	9.67 (8.72–10.62)
**2023**	4,153,408	448	10.79 (9.81 a 11.83)	76.71	11.71	7.55	6.09	8.93	11.08 (10.05–12.11)

Results showed as cases per 100,000 inhabitants. CI: confidence interval.

## Data Availability

Data are available upon reasonable request at the website of the Spanish Health Ministry: https://www.sanidad.gob.es/estadEstudios/estadisticas/estadisticas/estMinisterio/SolicitudCMBD.htm (accessed on 1 March 2025).
